# Dr. William Philip Spratling

**Published:** 1916-02

**Authors:** 


**Dr. William Philip Spratling,** P. & S. of Baltimore 1886, died suddenly at Wilska, Fla., Dec. 22, 1915. He was born in Chambers Co., Ala., in 1863, and received his education at Vanderbilt University and the Ala. State Polytechnic Institute. Shortly after graduating in medicine, he was admitted to the U. S. Marine Hospital Service (now U. S. Public Health Service), but resigned after a year and became Asst. Physician to the State Hospital for the Insane at Morristown, N. J., holding this position for five years. He then engaged in private practice in New York for about three years. From 1894 to 1908 he was Supt. of Craig Colony at Sonyea, N. Y., thus being the pioneer in this form of control of epileptics. He was an ex-president of the National Assn. for the Study of Epilepsy.

